# Babies Shoes

**Published:** 1885-12

**Authors:** 


### BABIES' SHOES.

"Tell me something about babies' shoes. How are they numbered?"

"No. 4 is the first shoe out of babyhood. No. 0 has a soft sole of white kid and pasteboard, and is the successor of the knit wool boots that are sold for babies in long dresses. Nos. 1, 2 and 3 have what is called the turned sole, sewed together on the wrong side and turned out. There are from four to five buttons on the side, and a black tassel is now fastened at the top in front. The latest is to have a vamp of French kid with calf uppers, or, what is still better, a half-boxed round toe, tipped with patent leather."

"Is there no change in the shape of children's shoes?"

"None. There can't well be because the sole must be sufficiently broad to stand the wear and tear. Square toes are preferred to round, because they allow freer devolopment to the toes. The spring heel, which was introduced nearty two years ago, is worn as early as two years of age, and has recently become fashionable for girls in their teens. It is nothing but a slip of leather inserted between the sole and that part of the shoe pressed by the wearer's heel. It is seldom that a smaller than a No. 8 is made with a regular heel, and that is on the common sense plan, low and broad. These and the large sizes have a higher top than has been usual for several years. Children would have better looking feet if they had wiser mothers, and the fault lies in the first shoes worn. One pair too short will ruin the feet, no matter how loose subsequent ones may be."—*New York Mail and Express*.

